# 

**Published:** 1890-01

**Authors:** 


					﻿Vol X.
JANUARY, 1890.
NO. 1.
JPHE
flOWKEOPATHlC pH^lClAJi,
A Monthly Journal of Homoeopathic Materia Medica
and Clinical Medicine.
“ He is a freeman whom truth makes free, and all are slaves beside.’ ’
"Seek the Truth; come whence it may, cost what it will."
EDITED AND PUBLISHED BY
WALTER ML JAMES, ML D.,
AND
GEORGE H. CLARK, ML D.
ASSISTED BY THE FOLLOWING CONTRIBUTORSi
E. T. Balch, M. D., Sooth Bend, Wash.
Clarence Willard ButlEh, M. D",
Montclair, N. J.
Edward Carleton, New York.
Daniel W. Clausen, M. D., Philada.
Stuart Close. M.D., Brooklyn.
Edward Cranch, M. D., Erie, Pa.
W. S. Gee, M. D.. Hyde Park, Ill.
Clarence C. Howard, MD., New York.
Samtoct. A. R'ttwra t.t.. M.D.. Reston.
Edmund J. Lee, M. D., Former Editor,
Philada.
C. F. Nichols. M. D.. Boston.
Mahlon ‘Preston, M. D., Norristown.
Geohge W. Sherbino, M. D., Abilene,
Texas.
Harlyn Hitchcock, M.D., New York.
A. McNeil, M. D., San Francisco.
Carleton Smith, M. D,, Philada.
PHILADELPHIA:
No. 1125 SPRUCE STREET.
zpposrcpQ ar.
ALFRED HEATH & CO., Sole Agents for Great Britain,
114 Ebury Street, Eaton Square.
Yearly Subscription, $2.50. invariably in advance. Single numbers, 25 cts.
Entered at the Philadelphia Peet-Office a* Second-Class Mail Matter.
				

